# Supplementary material for: Lactiplantibacillus argentoratensis and Candida tropicalis Isolated from the Gastrointestinal Tract of Fish Exhibited Inhibitory Effects against Pathogenic Bacteria of Nile Tilapia
Source: Vet Sci. 2023 Feb 7;10(2):129. doi: 10.3390/vetsci10020129 (PMC9958883; doi:10.3390/vetsci10020129)
Supplement: Supplementary file 1 [file vetsci-10-00129-s001.zip › Figure S1.pdf]

Figure S1: Plates showing clear inhibition zones of some probiotic isolates

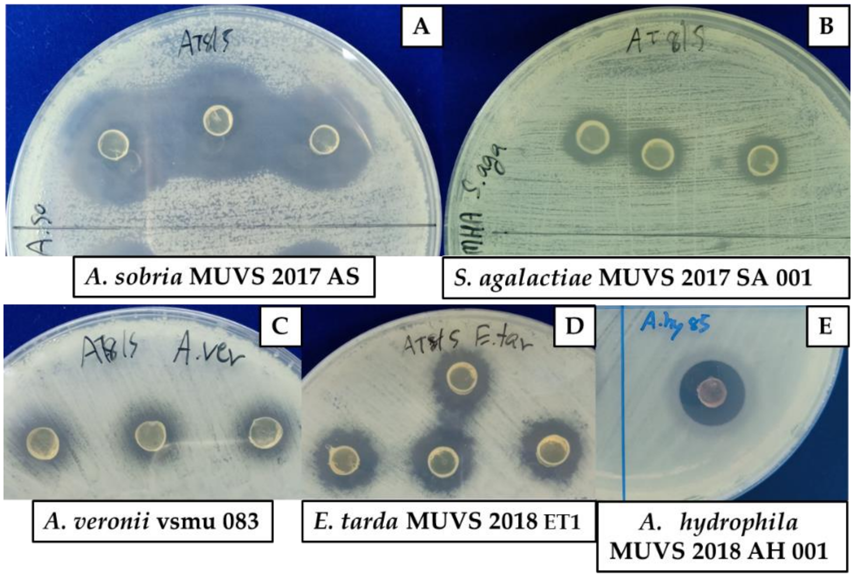

The clear inhibition zones of probiotic isolate AT8/5 against each of the five bacterial pathogens: (A) *A. sobria* MUVS 2017 AS; (B) *S. agalactiae* MUVS 2017 SA 001; (C) *A. veronii* vsmu 083; (D) *E. tarda* MUVS 2018 ET1; and (E) *A. hydrophila* MUVS 2018 AH 001.

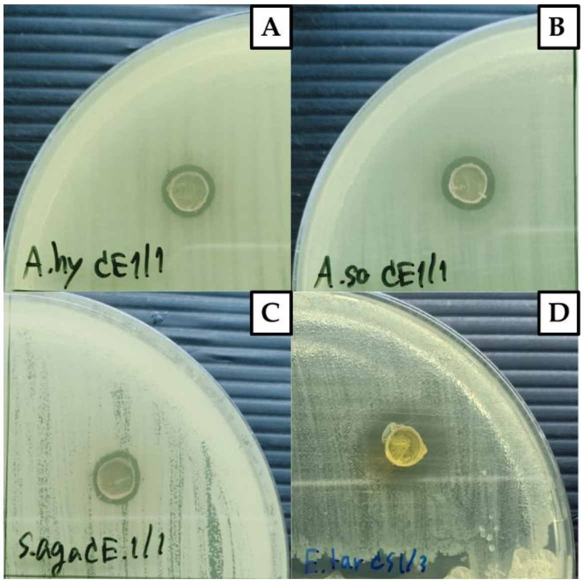

The antagonistic activity of some probiotic bacterial isolates against tested pathogens: (A) CE1/1 vs. *A. hydrophila* MUVS 2018 AH 001; (B) CE1/1 vs. *A. sobria* MUVS 2017 AS; (C) CE1/1 vs. *S. agalactiae* MUVS 2017 SA 001; and (D) CS1/3 vs. *E. tarda* MUVS 2018 ET1.

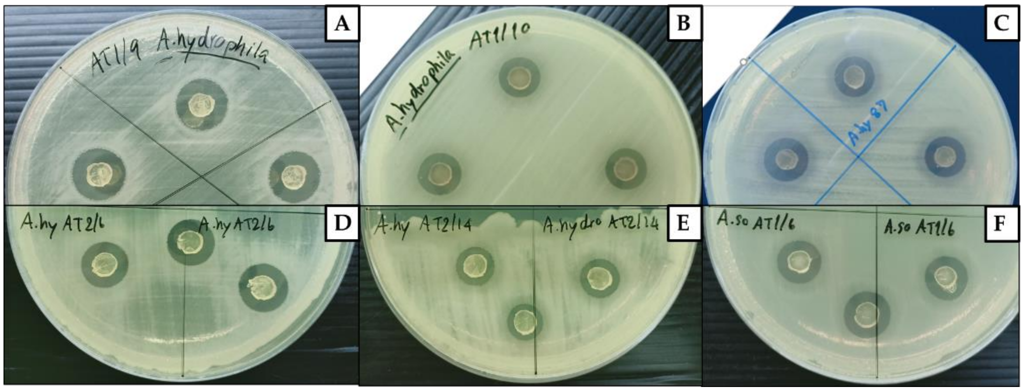

The antagonistic activity of some bacterial probiotic isolates against tested pathogenic bacteria: (A) AT1/9 vs. *A. hydrophila* MUVS 2018 AH 001; (B) AT1/10 vs. *A. hydrophila* MUVS 2018 AH 001; (C) AT8/7 vs. *A. hydrophila* MUVS 2018 AH 001; (D) AT2/6 vs. *A. hydrophila* MUVS 2018 AH 001; (E) AT2/14 vs. *A. hydrophila* MUVS 2018 AH 001; and (F) AT1/6 vs. *A. sobria* MUVS 2017 AS.

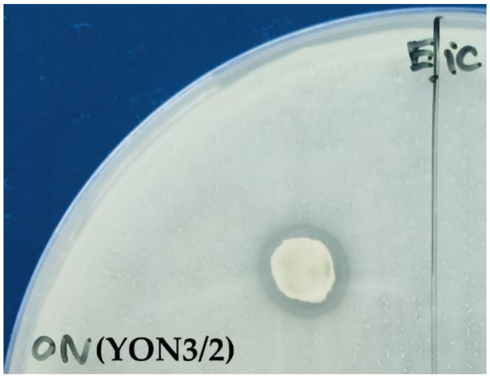

The zone of inhibition of yeast probiotic isolate YON3/2 against *E. ictaluri* 2010/12 EI.
