# Supplementary material for: Lactiplantibacillus argentoratensis and Candida tropicalis Isolated from the Gastrointestinal Tract of Fish Exhibited Inhibitory Effects against Pathogenic Bacteria of Nile Tilapia
Source: Vet Sci. 2023 Feb 7;10(2):129. doi: 10.3390/vetsci10020129 (PMC9958883; doi:10.3390/vetsci10020129)
Supplement: Supplementary file 1 [file vetsci-10-00129-s001.zip › Supplemental Table S9.pdf]

**Supplemental Table S9: The GenBank accession numbers and NCBI links of each identified probiotic isolate.**

| Isolate | Species | Accession number | NCBI link |
|---|---|---|---|
| CE1/1 | *Weissella paramesenteroides* | LC735573 | https://www.ncbi.nlm.nih.gov/nuccore/LC735573 |
| CS1/3 | *Weissella paramesenteroides* | LC735574 | https://www.ncbi.nlm.nih.gov/nuccore/LC735574 |
| AT8/5 | *Lactiplantibacillus argentoratensis* | LC735575 | https://www.ncbi.nlm.nih.gov/nuccore/LC735575 |
| YCS1/1 | *Kodamaea ohmeri* | LC735679 | https://www.ncbi.nlm.nih.gov/nuccore/LC735679 |
| YCS1/3 | *Kodamaea ohmeri* | LC735680 | https://www.ncbi.nlm.nih.gov/nuccore/LC735680 |
| YON3/2 | *Candida tropicalis* | LC735681 | https://www.ncbi.nlm.nih.gov/nuccore/LC735681 |
